# Primary spontaneous pneumomediastinum: 237 cases in a single-center experience over a 10-year period and assessment of factors related with recurrence

**DOI:** 10.1371/journal.pone.0289225

**Published:** 2023-07-26

**Authors:** Min Hyuk Yu, Jin Kyem Kim, Taeho Kim, Hong Seon Lee, Dong Kyu Kim

**Affiliations:** 1 Department of Radiology, The Armed Forces Capital Hospital, Seongnam, Korea; 2 Department of Radiology, Severance Hospital, Research Institute of Radiological Science, Yonsei University College of Medicine, Seodaemun-Gu, Seoul, Korea; University of Torino, ITALY

## Abstract

**Objective:**

To evaluate the precipitating factors and symptoms of primary spontaneous pneumomediastinum (PSPM) and to assess the factors related with recurrent spontaneous pneumomediastinum (RSPM).

**Methods:**

From 2010 to 2021, 237 PSPM patients were included in this retrospective study. Clinical information including in-hospital periods, morbidity, mortality, presenting symptoms, precipitating events, smoking, and asthma history was obtained. The patients with smoking history were subdivided into “ex-smoker” or “current smoker”. The severity of asthma was categorized into “mild intermittent”, “mild persistent”, “moderate persistent”, or “severe persistent”. During follow-up, patients with RSPM were classified into “recurrence” group and the others were into “no recurrence” group. Multivariate regression analysis was used to elucidate the associated factors with RSPM.

**Results:**

The mean age of study patients (men: women = 222: 15) was 23.4 years and mean period of hospital stay was 7.5 days. There was no mortality and morbidity. Most frequent symptom and precipitating factor were acute chest pain (n = 211, 89.0%) and cough (n = 72, 30.4%), respectively. RSPM occurred in 11 patients (4.6%). The proportion of patients with smoking (72.8% vs. 37.1%, *p* = 0.010) or asthma (81.8% vs. 39.8%, *p*<0.001) was significantly higher in “recurrence” group than “no recurrence” group. On multivariate analysis, asthma was the only factor associated with RSPM (mild intermittent/persistent, OR = 7.092, *p* = 0.047; moderate persistent, OR = 8.000, *p* = 0.011).

**Conclusion:**

PSPM is a benign disease with no morbidity and mortality. Asthma may be the associated factor with RSPM; thus, despite the low rate of recurrence, patients with asthma should be informed about the chance of RSPM.

## Introduction

Pneumomediastinum, defined as the presence of free-air within the mediastinum, can be divided into two types: spontaneous or secondary. Secondary pneumomediastinum may commonly occur in situations which can damage the esophagus or airway such as trauma, surgery, and other interventions, or by gas-forming mediastinal infections. In contrast, primary spontaneous pneumomediastinum (PSPM) is a diagnosis of exclusion and is rare, but mainly benign disease [1].

Although occasionally obvious precipitating factors cannot be identified in patients with PSPM, previous studies reported that there were benign precipitating factors including asthma, smoking, coughing, vomiting, training, or etc. for PSPM [2]. However, despite previous studies on PSPM [3–6], the disease was relatively poorly understood because most previous research have been conducted in fewer than 100 patients with PSPM, and research including over 100 patients is scarce [7]. In addition, since recurrent spontaneous pneumomediastinum (RSPM) is far rare, not much published information was available on RSPM and furthermore, not many studies have evaluated whether there was associated factor with RSPM [8, 9].

Therefore, the aim of this study was 1) to evaluate the precipitating factors and symptoms of PSPM in a relatively large sample size at a single-center with over a 10-year period and 2) to assess the factors related with RSPM.

## Materials and methods

### Study population

This single-center retrospective study was approved by our institutional review board, which waived the requirement for informed consent.

**Fig 1:**
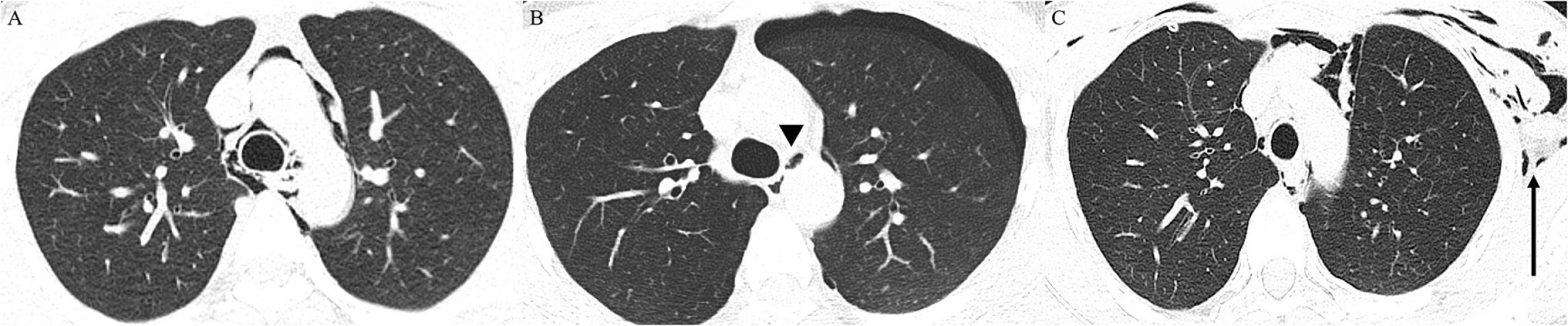
Representative cases of primary spontaneous pneumomediastinum (PSPM) on chest computed tomography (CT). (A) pneumomediastinum without other combined complication, (B) pneumomediastinum (arrowhead) with left pneumothorax, and (C) pneumomediastinum with subcutaneous emphysema (arrow) at anterior chest wall (left > right).

From January 2010 to December 2021, 237 patients with their first diagnosed PSPM were eligible for inclusion in this study. All the study patients underwent unenhanced or contrast-enhanced chest computed tomography (CT) (Fig 1) and underwent chest radiographs on a daily basis until they were discharged. During the follow-up period, patients with recurrent spontaneous pneumomediastinum were classified into “recurrence” group and those without recurrent pneumomediastinum were classified into “no recurrence” group.

### Clinical records

Clinical information including age, sex, period of hospital stays, morbidity and mortality, presenting symptoms, combined thoracic abnormality (e.g., subcutaneous emphysema or pneumothorax), presence of precipitating events, smoking history, and asthma history was obtained from the electronic medical charts. Then, the patients with smoking history were subdivided into “ex-smoker” or “current smoker”. In patients with asthma, the severity of asthma was categorized into “mild intermittent”, “mild persistent”, “moderate persistent”, or “severe persistent”, based on daytime symptoms, nighttime symptoms and lung function [10]. During the follow-up period, it was checked whether there were patients with RSPM.

### Statistical analysis

Continuous variables are expressed as mean ± standard deviation (SD) or as median with interquartile range (IQR). Continuous variables were compared by using one-way analysis of variance, while categorical variables were compared by using Fisher exact or Chi-square tests. Univariate and multivariate logistic regression analyses were used to identify the risk factors for recurrent pneumomediastinum. Factors significantly associated with recurrence (*p* < 0.1) in the univariate analysis were included in the multivariate analysis. Outcomes are expressed as odds ratio (OR) and 95% confidence intervals (CIs). All statistical analyses were performed with SPSS 27.0 for Windows (SPSS Inc., IBM) and *P* < 0.05 were considered as statistically significant.

## Results

### Baseline characteristics of study population

There was no diagnosed specific disease or injury in the esophagus and airway based on imaging studies including chest CT and/or fluoroscopic upper gastrointestinal studies.

The mean and median age of the 237 patients with PSPM was 23.4 ± 3.4 years and 22 years (IQR: 21–25 years), respectively. The mean period of hospital stay was 7.5 ± 2.6 days (median: 8 days, IQR: 5–10 days). There was no mortality and morbidity, and any treatment such as oxygen inhalation or antibiotics did not affect hospital course.

### Clinical findings

Most of study population was men (93.7%) (men: women = 222: 15). Most frequent symptom was acute chest pain (n = 211, 89.0%), followed by acute neck pain (n = 123, 51.9%), and dyspnea (n = 78, 32.9%). The 1^st^ precipitating factor was cough (n = 72, 30.4%), followed by upper respiratory infection (n = 66, 27.8%), physical exertion (n = 56, 23.6%), and retching/vomiting (n = 34, 14.3%). There was no precipitating factor in 29 of 237 patients (12.2%). Of the 92 patients with smoking history (38.8%), 66 patients were ex-smokers and 26 patients were current smokers. In 99 patients with asthma history (41.8%), 88 patients had mild intermittent or mild persistent asthma, and 11 patients had moderate persistent asthma. There was no patient with severe persistent asthma. Subcutaneous emphysema and pneumothorax was combined in 6 patients (2.5%) and 9 patients (3.8%), respectively (Table 1). In cases of pneumothorax, the presence of pneumomediastinum did not change the standard treatment.

**Table 1:** Baseline characteristics of the study patients.

Characteristic	Recurrence	No recurrence	*p* value	Total
No. of patients	11	226		237
Age (years), median (IQR)	23 (22–27)	22 (20–25)	0.151	22 (21–25)
Sex, n (%)			0.098	
Men	9 (81.8)	213 (94.2)		222 (93.7)
Women	2 (18.2)	13 (5.8)		15 (6.3)
Presenting symptom, n (%)				
Chest pain	9 (81.8)	202 (89.4)	0.364	211 (89.0)
Neck pain	6 (54.5)	117 (51.8)	0.857	123 (51.9)
Dyspnea	3 (27.3)	75 (33.2)	0.684	78 (32.9)
Precipitating event, n (%)				
Upper respiratory infection	2 (18.2)	64 (28.3)	0.464	66 (27.8)
Cough	2 (18.2)	70 (31.0)	0.368	72 (30.4)
Physical exertion	3 (27.3)	53 (23.5)	0.771	56 (23.6)
Retching / Vomiting	3 (27.3)	31 (13.7)	0.210	34 (14.3)
Smoking history, n (%)			0.010	
Non-smoker	3 (27.3)	142 (62.8)		145 (61.2)
Ex-smoker	4 (36.4)	62 (27.4)		66 (27.8)
Current smoker	4 (36.4)	22 (9.7)		26 (11.0)
Asthma history, n (%)*			< 0.001	
None	2 (18.2)	136 (60.2)		138 (58.2)
Mild intermittent / Mild persistent	6 (54.5)	82 (36.3)		88 (37.1)
Moderate persistent	3 (27.3)	8 (3.5)		11 (4.6)
Combined thoracic finding, n (%)				
Subcutaneous emphysema	1 (9.1)	5 (2.2)	0.156	6 (2.5)
Pneumothorax	1 (9.1)	8 (3.5)	0.347	9 (3.8)

IQR = interquartile range

* The severity of asthma was categorized into “mild intermittent”, “mild persistent”, “moderate persistent”, or “severe persistent”, based on daytime symptoms, nighttime symptoms, and lung function. There was no patient who had severe persistent asthma.

### Factors associated with recurrent spontaneous pneumomediastinum

During the mean follow-up period of 48.6 ± 30.9 months, RSPM occurred in 11 patients (4.6%). The proportion of smokers (72.8% vs. 37.1%) and current smokers (36.4% vs. 9.7%) was significantly higher in “recurrence” group than “no recurrence” group (*p* = 0.010). Furthermore, not only the proportion of patients with asthma (81.8% vs. 39.8%), but that of patients with moderate persistent asthma (27.3% vs. 3.5%) was also significantly higher in “recurrence” group than “no recurrence” group (*p* < 0.001). There was no significant difference in age, sex, presenting symptoms, precipitating events, and combined thoracic disease between two groups (Table 1). One patient with pneumothorax in the recurrence group had no pneumothorax during the first episode.

Table 2 showed associations of selected factors with RSPM. On univariate analysis, current smoker (OR = 5.051, 95% CI = 0.922–35.455, *p* = 0.060) and asthma (mild intermittent/mild persistent asthma, OR = 4.082, 95% CI = 1.001–9.130, *p* = 0.038; moderate persistent asthma, OR = 6.061. 95% CI = 1.017–35.714, *p* = 0.001) were the related factors to recurrence. However, multivariate analysis showed that asthma was the only factor associated with a higher risk of RSPM (mild intermittent/mild persistent asthma, OR = 7.092, 95% CI = 0.925–29.619, *p* = 0.047; moderate persistent asthma, OR = 8.000, 95% CI = 1.000–40.000, *p* = 0.011).

**Table 2:** Univariate and multivariate analysis for predictors of recurrent pneumomediastinum.

Variables	Univariate Analysis			Multivariate Analysis		
	OR	95% CI	*p* value	OR	95% CI	*p* value
Age	3.315	0.478–23.978	0.232			
Sex	5.988	0.641–35.556	0.216			
Pneumothorax	3.448	0.484–27.478	0.339			
Subcutaneous emphysema	9.434	1.826–111.111	0.466			
Smoking						
Non-smoker	Reference					
Ex-smoker	3.937	0.506–30.303	0.191			
Current smoker	5.051	0.922–35.455	0.060	6.452	0.700–37.037	0.105
Asthma						
None	Reference					
Mild intermittent / Mild persistent	4.082	1.001–8.130	0.038	7.092	0.925–29.619	0.047
Moderate persistent	6.061	1.017–35.714	0.001	8.000	1.000–40.000	0.011

OR = Odds ratio, CI: Confidence interval

## Discussion

In the present study, all the 237 patients with PSPM showed benign clinical course without morbidity nor mortality. Most frequent symptom was acute chest pain (89.0%), followed by acute neck pain (51.9%), and dyspnea (32.9%). The 1^st^ precipitating factor was cough (30.4%), followed by upper respiratory infection (27.8%), physical exertion (23.6%), and retching/vomiting (14.3%). During the mean follow-up period of 48.6 ± 30.9 months, RSPM occurred in 11 patients (4.6%). On multivariate regression analysis, asthma was the only associated factor with RSPM (mild intermittent/mild persistent asthma, OR = 7.092, 95% CI = 0.925–29.619, *p* = 0.047; moderate persistent asthma, OR = 8.000, 95% CI = 1.000–40.000, *p* = 0.011).

Though there have been previous studies regarding PSPM, the present study with relatively large sample size was conducted to improve the understanding of PSPM and RSPM, since research on over 100 patients with pneumomediastinum is scarce. The results of our single-center study are in many ways consistent with previous reports. First, our result shows that PSPM is a self-limited and benign disease; thus, specific treatment may not be required in PSPM patients without concurrent pneumothorax. Second, the mean age was 23.4 ± 3.4 years (median: 22 years, IQR: 21–25 years) and the proportion of men was overwhelmingly high (222/237, 93.7%), concordant with the existing knowledge that PSPM generally affected young adult males [11, 12]. Third, chest pain was the most common symptom and common precipitating events included cough, physical exertion, and retching/vomiting. Furthermore, smoking and asthma were common histories in patients with PSPM [7].

In this study, the recurrence rate of spontaneous pneumomediastinum was 4.6%, comparable to the reported PSPM recurrence rates of 0–4.5% in previous case series studies [4, 6, 13–16]. However, since RSPM was rare disease even more than PSPM, there was few research on cause or related factors of RSPM. Our results showed that the proportion of patients with smoking (72.8% vs. 37.1%, *p* = 0.010) or asthma (81.8% vs. 39.8%, *p* < 0.001) was significantly higher in “recurrence” group than “no recurrence” group. However, on multivariate analysis, asthma was the only associated factor with recurrence (mild intermittent/mild persistent asthma, OR = 7.092, *p* = 0.047; moderate persistent asthma, OR = 8.000, *p* = 0.011). Asthma exacerbation has been known as one of the most prominent risk factors for PSPM and PSPM has been reported both as a sign of a first asthma attack and as a consequence of an asthma exacerbation. Furthermore, PSPM may be associated with poorly-controlled asthma [17, 18]. Therefore, both the clinical suspicion of asthma when spontaneous pneumomediastinum occurs and that of spontaneous pneumomediastinum when acute chest pain occurs in patients with asthma are essential.

There are some limitations in this study. The main limitation is the small sample size. There were only 11 patients included in the recurrent group. To validate the results that asthma and smoking are significantly associated factors with RSPM, studies involving more RSPM patients are needed. Second, the present study was conducted based on retrospective design, and selection bias could affect the results. Third, since poorly-controlled asthma is known to be associated with spontaneous pneumomediastinum, it may be necessary to check whether asthma is well controlled by medication or not; however, it is difficult to confirm because this clinical information was not accurately recorded in the electronic medical charts. Further research is needed on these areas, and an improved understanding of associated factors with PSPM and RSPM can lead to more efficient management of patients with spontaneous pneumomediastinum.

In conclusion, PSPM is a self-limited and benign disease and specific treatment may not be required if there is not concurrent pneumothorax. Furthermore, patients with asthma should be informed about the chance of recurrence despite the low rate of recurrence, since the asthma may be the associated factor with RSPM.
